# Etiologies and Treatment Burden in Adult Patients with Pure Red Cell Aplasia: A Single-Center Experience and Review of Literature

**DOI:** 10.1155/2020/4812759

**Published:** 2020-03-15

**Authors:** Pimjai Niparuck, Wasana Kanoksil, Pathawut Wacharapornin, Pichika Chantrathammachart, Sarinya Boongird

**Affiliations:** ^1^Division of Hematology, Department of Medicine, Ramathibodi Hospital, Mahidol University, Bangkok, Thailand; ^2^Department of Pathology, Ramathibodi Hospital, Mahidol University, Bangkok, Thailand; ^3^Division of Nephrology, Department of Medicine, Ramathibodi Hospital, Mahidol University, Bangkok, Thailand

## Abstract

**Background:**

Pure red cell aplasia (PRCA) is less common blood disorder; the causes and the treatments of PRCA are varied.

**Methods:**

We conducted a retrospective study during January 2010–December 2017, to explore the etiologies and to evaluate the response and treatment burden in adult patients with PRCA.

**Results:**

Of 32 PRCA patients, median age was 57 years (18–90 years). Median hemoglobin level and reticulocyte count at the time of diagnosis were 5.6 g/dL (3.3–7.3 g/dL) and 0.3% (0.1–0.7%), respectively. Median time to hematologic recovery was 12 weeks (3–72 weeks), and median number of red blood cell transfusion (RBC) was 20 units (4–100 units). Causes of PRCA were erythropoiesis-stimulating agent (ESA) (47%), parvovirus B19 infection (19%), thymoma (13%), zidovudine (6%), primary autoimmune PRCA (6%), Kaposi's sarcoma (3%), systemic lupus erythematosus (3%), and ABO-mismatched stem cell transplantation (3%). Only 9 out of 24 treated patients achieved hematologic response within 8 weeks of treatment. Intravenous immunoglobulin therapy provided 100% response rate in patients with parvovirus B19-associated PRCA and primary autoimmune PRCA. Low response rate was found in patients receiving immunosuppressants and chemotherapy for the treatment of ESA and thymoma-associated PRCA, respectively.

**Conclusions:**

Treatment outcome of PRCA depended upon the causes and the types of treatment, and the burden of RBC transfusion was very high in patients with ESA and thymoma-associated PRCA.

## 1. Introduction

Pure red cell aplasia (PRCA) is a rare but devastating blood disorder presenting with normocytic anemia, severe reticulocytopenia, and markedly decreased number of erythroid precursors from the bone marrow (BM). Acquired PRCA may be caused by parvovirus B19, human immunodeficiency virus (HIV), thymoma, solid tumor, lymphoproliferative disorder, antierythropoietin (EPO) antibody, systemic lupus erythematosus (SLE), rheumatoid arthritis (RA), pregnancy, and major ABO-mismatched stem cell transplantation or primary autoimmune PRCA [1, 2]. In addition, isoniazide (INH), azathioprine (AZA), mycophenolate mofetil (MMF), tacrolimus (FK-506), zidovudine (AZT), lamivudine (3TC), and allopurinol are also reported as the cause of drug-mediated PRCA. Treatment of PRCA depends on the etiologies, and immunosuppressive therapies remain the mainstay of treatment for antibody-mediated PRCA. Nevertheless, the responses of primary or secondary immune-mediated PRCA to immunosuppressive therapies widely vary according to the protocols. Response rate of corticosteroids (Cs) and cyclophosphamide (Cy) were quite low, ranging from 30–60% and 20–40%, respectively. However, cyclosporin A (CSA) induces higher response rate, approximately 65–85% of cases [1–3]. Other armamentaria for PRCA treatments are intravenous immunoglobulin (IvIg), antithymocyte globulin (ATG), and anti-CD20 (Mabthera); however, the treatment outcomes are scarce. PRCA patients require frequent red blood cell transfusion (RBCT) while awaiting for resolution of PRCA. Since there were few studies or case series which described the causes of adult patients with PRCA in Asian countries [4–8], we, therefore, conducted this retrospective study to explore the etiologies of PRCA and to evaluate the treatment response and the burden of treatment in adult patients diagnosed with PRCA.

## 2. Materials and Methods

We enrolled adult patients diagnosed with PRCA during January 2010 to December 2017 at Ramathibodi Hospital. The diagnosis of PRCA is defined as anemia with severe reticulocytopenia and the number of erythroid progenitor cells in BM <5%. The data including age, gender, disease, medication, onset of PRCA, number of pack red cell transfusion, laboratory results, and response of treatment were collected from patient medical records. All histopathological slides were reviewed, and the diagnosis of PRCA was confirmed by an experienced hematopathologist. Patients aged <15 years were excluded from the study.

## 3. Study Design and End Points

### 3.1. Primary End Points

The objectives of the study were to identify the causes of PRCA and to analyze the rate of hematologic response in adult PRCA patients receiving various regimens of immunosuppressant or other treatments. Hematologic response was defined as an increase in hemoglobin level, reticulocyte count >1% and becoming RBC transfusion independent. Treatment failure was defined as no hematologic response within 8 weeks after treatment with immunosuppressive or other therapies.

### 3.2. Secondary End Points

To evaluate the treatment burden in adult patients diagnosed with PRCA.

## 4. Results

A total of 32 patients were identified, and median age was 57 years (range: 18–90 years). Median hemoglobin (Hb) level and reticulocyte count at the time of diagnosis were 5.6 g/dL (3.3–7.3 g/dL) and 0.3% (0.1–0.7%), respectively. The causes of PRCA were erythropoiesis-stimulating agent (ESA) (15 patients), parvovirus B19 infection (6 patients), thymoma (4 patients), AZT (2 patients), Kaposi's sarcoma (1 patient), SLE (1 patient), ABO-mismatched stem cell transplantation (1 patient), and primary autoimmune PRCA (2 patients). Twenty-four patients (75%) received treatment, the remaining 8 patients, all of whom were diagnosed with ESA-induced PRCA and received only supportive treatment with RBCT. In the entire study population, the median time to achieve hematologic recovery were 12 weeks (3–72 weeks), and the median number of RBCT was 20 units (4–100 units). Hematologic response within 8 weeks of treatment was achieved in 9 out of 24 patients (38%) (Table 1). Of 9 patients, 7 received IvIg therapy, five had parvovius B19, and two had primary autoimmune PRCA. The remaining 2 patients diagnosed with HIV achieved hematologic response after cessation of AZT. Treatment failure was found in 15 out of 24 treated patients.

In the group of ESA-induced PRCA, all 15 patients previously received epoetin alfa therapy after developing anemia associated with chronic kidney disease (CKD) stages 3–5 (13 patients), myelodysplastic syndrome (1 patient), and anemia of inflammation (1 patient). Twelve out of fifteen patients (80%) were treated with biosimilar epoetin alfa products. A bone marrow study was performed in all patients, whereas antibody to anti-EPO was performed in 10 out of 15 patients. The median time to development of PRCA after treatment with ESA was 10 months (5–21 months). Of 15 patients with ESA-induced PRCA, 3, 1, 1, 1, and 1 were treated with combined immunosuppressive therapies, kidney transplantation, oxymetholone, Mabthera, and IvIg, respectively. The remaining 8 patients received only RBCT. Three patients receiving combined immunosuppressive therapy (CSA/prednisolone ± cyclophosphamide) did not achieve response, and the hemoglobin levels were improved and became nontransfusion-dependent after treatment with immunosuppressants for 13–16 months. In contrast, the Hb level was recovered in patients treated with IvIg, Mabthera, or kidney transplantation at 12–13 weeks of therapy, but there was no response in patients treated with oxymetholone. Patients receiving kidney transplant was previously treated with 3-month period of combined immunosuppressive therapy (prednisolone, CSA, and cyclophosphamide) for PRCA, but did not achieve hematologic response. Four out of eight ESA-induced PRCA patients receiving supportive therapy achieved spontaneous hematologic recovery after 1 year (12–18 months), and the remaining 4 patients had been followed up by our hematologists for 4–5 months before returning to their nephrologists for long term follow-up at other hospitals. The median number of RBCT in patients with ESA-induced PRCA was 30 units (8–52 units); 41 (12–52 units) and 23 units (8–50 units) of RBCT were given in patients receiving supportive therapy and specific therapies, respectively.

Three CKD patients with ESA-induced PRCA who achieved spontaneous hematologic recovery were successfully rechallenged erythropoietin beta therapy for treatment of their anemia from CKD. Erythropoietin beta was resumed after their first diagnosis of PRCA for 5 years (1 patient) and 2 years (2 patients), and all 3 patients were not retested anti-EPO antibodies before starting erythropoietin beta therapy. Hb levels increased from 7.5–8.2 g/dL to 9.5–11.5 g/dL after erythropoietin beta therapy, and there was no evidence of recurrent PRCA after initiating this treatment for 13–20 months.

In the group of parvovirus B19-induced PRCA, 3 patients had HIV infection with CD4 cell count ranging from 18–217 cell/mm^3^, and the remaining 3 patients were kidney transplant recipients. Two and one HIV patients had parvovirus B19 infection at the time of diagnosis of HIV infection and at 10 months of antiretroviral therapy, respectively. Giant pronormoblast was found in the BM tissues of all patients, and parvovirus B19 viremia was detected in 5 out of 6 patients. IvIg was given in all 6 patients, and the rate of response was 100% with a median time to response of 3.5 weeks (3–12 weeks). The median number of RBCT in these patients was 4 units (2–6 units).

The causes of PRCA in the other 3 HIV patients were AZT induced (2 patients) and Kaposi's sarcoma (1 patient). The CD4 cell count in HIV patients with AZT and Kaposi's sarcoma-induced PRCA were 199–271 and 450 cell/mm^3^, respectively. Chemotherapy has yielded hematologic response at 3 months of therapy in one patient with Kaposi's sarcoma. In patients with AZT-induced PRCA, they achieved hematologic response after cessation of AZT for 3–4 weeks. One of two patients who developed AZT-induced PRCA was initially diagnosed with parvovirus B19 infection because her BM examination demonstrated erythroid progenitor cells <5% with few giant pronormoblast and her long duration of AZT treatment (10 years) without prior hematologic problem. However, further investigation revealed no evidence of parvovirus B19 viremia, and the patient did not respond to initial IvIg therapy. BM study was reperformed at 2 months of IvIg therapy and again found decreased erythroid progenitor cells. We decided to discontinue AZT, and surprisingly her Hb was improved and became nontransfusion-dependent within 4 weeks.

In patients diagnosed with malignant thymoma (4 patients), 2 patients had PRCA at the time of diagnosis of thymoma, while one patient developed PRCA after progressive disease (7 months after diagnosis of thymoma) and the another one had PRCA at the time of disease relapse (3 years). Three and one patients were treated with six cycles of chemotherapy alone and thymectomy alone, respectively. Combined immunosuppressive therapy was given in 3 patients with unresectable tumors who did not achieve hematologic recovery after completion of 6 cycles of chemotherapy, and only 2 patients achieved hematologic response after treatment with CSA with corticosteroids, and the response was seen at 7 and 8.5 months after starting chemotherapy. The remaining 2 patients had progressive disease and died from disease progression (1 patient) and *E.coli* septicemia (1 patient). The median number of RBCT in thymoma-induced PRCA was 50 units (20–100 units).

There were 2 middle-aged female patients diagnosed with primary autoimmune PRCA in this study. They were treated with IvIg and achieved hematologic response within 4–6 weeks. The median number of RBCT was 8 units (6–10 units). Relapsed PRCA was observed in 1 patient at 2 years of IvIg therapy, and she was treated with IvIg combined with CSA therapy and achieved a second hematologic response at 4 weeks after treatment. CSA therapy was continued for 3 years after achieving second hematologic response.

## 5. Discussion

We found only 32 patients diagnosed with PRCA during the study period which occurred in a small number of patients. The most common causes of PRCA were ESA-induced antibody-mediated PRCA and parvovirus B19 infection. Except AZT-induced PRCA, there was no occurrence of INH, AZA, MMF, FK-506, 3TC, antiepileptic drug, or allopurinol-induced PRCA in this study [1]. PRCA from hematologic malignancies was also not found. ESA-induced PRCA was frequently seen in the study since there were many brands of erythropoietin available in our country, and the issues that the physicians should be considered before choosing the type of erythropoietin therapy were the structure of erythropoietin (epoetin molecules differing in the carbohydrate structure), pharmacokinetic, pharmacodynamics, potency, immunogenicity, safety, purity, and also the coated rubber stoppers [9]. Although the incidence of ESA-induced PRCA was very low, the complication and the treatment burden in patients developed PRCA were extremely high, and all patients with ESA-induced PRCA required multiple red cell transfusions while waiting for achieving a hematologic recovery. In this study, the spontaneous hematologic recovery was found in ESA- and AZT-induced PRCA; however, a longer time to achieve hematologic recovery was seen in patients with ESA-induced PRCA. The patients receiving immunosuppressant or other therapies for treatment of ESA-induced PRCA required RBCT lesser than those receiving only supportive therapy, even the treatment failure was found in our patients receiving combined immunosuppressants. Mabthera® or IvIg® induced rapid hematologic recovery in these patients; therefore, Mabthera or IvIg might be the treatment options for ESA-induced PRCA. Nevertheless, the number of ESA-induced PRCA patients receiving treatment in the study was very small to address the initial treatment for ESA-induced PRCA. All HIV patients with parvovirus B19 infection had CD4 cell counts <250 cell/mm^3^ which the result was similar to the previous study [10]. PRCA from parvovirus B19 infection in both HIV and non-HIV patients had a very good response to IvIg therapy. However, the response of treatment with chemotherapy in patients with thymoma-associated PRCA was very low. All thymoma patients needed to be treated PRCA with immunosuppressive therapy after completion of chemotherapy which induced hematologic response in 67% of cases. According to a previous study, the response rate of thymectomy for thymoma-associated PRCA was very low, whereas the authors described the treatment with antithymocyte globulin (ATG) plus CSA was shown to be an effective immunosuppressant against thymoma-induced PRCA [11]. In this study, only 2 patients were diagnosed with primary autoimmune PRCA, and they achieved hematologic response from IvIg therapy, while the study from Japan illustrated that CSA induced 75% of CR/PR in idiopathic PRCA patients [7].

## 6. Conclusions

PRCA was an uncommon blood disorder which the common causes of PRCA in the study were ESA, parvovirus B19 infection, and thymoma. Favorable treatment response occurred in groups of parvovirus B 19 infection, drug- (non-ESA-) induced PRCA, and idiopathic PRCA. However, unfavorable outcome was found in groups of ESA and thymoma-induced PRCA, even receiving immunosuppressants or chemotherapy, respectively.

## Figures and Tables

**Table 1:** Causes and treatment outcomes of adult patients with PRCA.

Cause of PRCA/gender	Median time to develop PRCA from diagnosis/treatment (Mo)	Treatment (*N*)	Response rate within 2 Mo (%)	Median number of PRCT	Time to hematologic recovery (wk)	Relapse rate (%)
Erythropoietin/F:M = 9 : 6	10 (5–21)	Immunosuppressant (*N* = 4)	0	27 (8–50)	52 (40–64)	0
		Other therapies (*N* = 4)	0	14 (8–32)	13 (12–14)	0
		Supportive treatment (*N* = 8)	—	40 (34–52)	68 (52–72)	0

Parvovirus B19 infection:					
(i) HIV infection/F:M = 0 : 3	6 (0–10)	IvIg (*N* = 3)	100	4 (2–6)	3 (3–4)	0
(ii) Kidney transplantation/F:M = 1 : 2	4 (1–5)	IvIg (*N* = 3)	100	4 (4–5)	4 (3–12)	0

Thymoma/F:M = 3 : 1	3.5 (0–36)	Thymectomy (*N* = 1, type B3)	0	100	—	—
		Chemotherapy and immunosuppressive therapy (*N* = 3, type *B*1 = 2, *B*3 = 1)	0	30 (20–50)	31 (28–34)	0
		Thymectomy/chemotherapy (*N* = 0)	—	—	—	—

Zidovudine (AZT)/F:M = 1 : 1	84 (48–120)	Cessation of AZT (*N* = 2)	100	30 (10–50)	3.5 (3–4)	0

Primary autoimmune PRCA/F:M = 2 : 0	—	IvIg (*N* = 2)	100	8 (6–10)	5 (4–6)	50

Systemic lupus nephritis/F:M = 1 : 0	156	Cellcept/danazol/pred/Mabthera	0	30	132	0

Kaposi sarcoma/F:M = 0 : 1	60	Chemotherapy	0	6	12	0

Transplant (ABO mismatch)/F:M = 1 : 0	4	Cyclosporin A/rEPO/Androlic/Velcade	0	75	104	0

## Data Availability

The data used to support the findings of this study are available from the corresponding author upon request.
